# The role of activated partial thrombin time in mediating the impact of poorly glycemic control on diabetic peripheral neuropathy in patients with type 2 diabetes mellitus

**DOI:** 10.3389/fendo.2025.1501323

**Published:** 2025-01-23

**Authors:** Hui Zhang, Minghui Chen, Lijie Sun, Wenwen Zhu, Tong Niu, Huzaifa Fareeduddin Mohammmed Farooqui, Hongxiao Wang, Bing Song, Jumei Wang, Haoqiang Zhang

**Affiliations:** ^1^ Henan Key Laboratory of Rare Diseases, Endocrinology and Metabolism Center, The First Affiliated Hospital, and College of Clinical Medicine of Henan University of Science and Technology, Luoyang, China; ^2^ Department of Endocrinology, Institute of Endocrine and Metabolic Diseases, Centre for Leading Medicine and Advanced Technologies of IHM, The First Affiliated Hospital of USTC, Division of Life Sciences and Medicine, University of Science and Technology of China, Hefei, China; ^3^ Department of Endocrinology, Huzhou Central Hospital, Affiliated Central Hospital of Huzhou University, Fifth School of Clinical Medicine of Zhejiang Chinese Medical University, Huzhou, China; ^4^ Affiliated Zhongda Hospital of Southeast University, Nanjing, China; ^5^ Department of Endocrinology, The First Affiliated Hospital of Jinzhou Medical University, Jinzhou, China

**Keywords:** activated partial thrombin time, diabetic peripheral neuropathy, type 2 diabetes mellitus, hyperglycemia, nerve conduction velocity

## Abstract

**Aim:**

This study aims to investigate the role of activated partial thrombin time (APTT) as a potential mediator in the relationship between suboptimal glycemic control and diabetic peripheral neuropathy (DPN) in individuals with type 2 diabetes mellitus (T2DM).

**Methods:**

A total of 183 T2DM patients were enrolled in this study. Comprehensive clinical data, including coagulation parameters and nerve conduction velocity, were collected and compared between patients with and without DPN. Subsequent correlation and regression analyses were conducted to explore the associations among APTT, HbA1c levels, and nerve conduction velocities. Moreover, mediation analyses were performed to evaluate the total, direct, and indirect effects of HbA1c on specific nerve conduction velocities, with APTT serving as a mediator.

**Results:**

In comparison to 101 patients without DPN, 82 patients with DPN exhibited significantly elevated levels of HbA1c and decreased levels of APTT. Notably, levels of APTT and HbA1c were correlated with conduction velocities of Tibial nerve motor fibers, as well as sensory fibers of the Ulnar nerve, Median nerve, and Sural nerve. Furthermore, both elevated HbA1c and decreased APTT were identified as risk factors for DPN in T2DM individuals. Mediation analysis showed that APTT mediated the indirect effect of HbA1c on the conduction velocities of sensory fibers in both the ulnar nerve and sural nerve (95% CI: -0.3448, -0.0135; -0.3523, -0.0180). APTT mediated the relationship between HbA1c and the conduction velocities of sensory fibers in the ulnar nerve or sural nerve by 34.66% or 22.03%, respectively.

**Conclusions:**

In patients with T2DM, uncontrolled HbA1c and shorter APTT emerges as risk factors for DPN. Additionally, the effect of increased HbA1c upon DPN, especially for influenced conduction velocities of sensory fibers in both the ulnar nerve and sural nerve may partly medicated by decreased APTT.

## 1 Introduction

One of the key characteristics of patients with diabetes mellitus is chronic hyperglycemia, which is closely associated with the onset and progression of various chronic complications, including diabetic peripheral neuropathy (DPN) (1, 2). Additionally, coagulation disorders is commonly occurrence in patients with diabetes mellitus (3). Fibrinogen and D-dimer are vital components associated with coagulation function and are commonly assessed in clinical settings to evaluate patients’ coagulation abilities (4, 5). The plasma fibrinogen may be a contributing factor for diabetic neuropathy and could be used as an indicator in the early screening and diagnosis of peripheral neuropathy in patients with type 2 diabetes mellitus (T2DM) (6). In addition to diabetes itself, patients with T2DM who have chronic complications tend to exhibit higher levels of fibrinogen and D-dimer in their plasma compared to those without complications, with elevated fibrinogen being one of the independent risk factors for these complications (7). Additionally, elevated levels of D-Dimer were also observed in patients with complications as compared to those without diabetic complications (8). D-dimer levels in type 1 and type 2 diabetic children and adolescents are relate to microvascular complications (9), with showed links with DPN. Indeed, increased plasma D-dimer levels may be a promising indicator for DPN in patients with T2DM (10).

Our previous research has found a close correlation between blood plasma glucose and DPN (11). An analysis of clots formed from fibrinogen extracted from individuals with T2DM and controls revealed that samples from those with T2DM displayed denser and less porous clots (12). This study suggesting a correlation between hyperglycemia and coagulation dysfunction. Glycation is a non-enzymatic reaction occurring in a chronic hyperglycemic state. This phenomenon can be attributed to increased glycation of fibrinogen in diabetes. A study may have indirectly confirmed this point: when plasma glucose levels were effectively controlled, the coagulation function of diabetic patients was improved (13). Alterations in fibrinogen function entail more complexity than mere concentration changes. Those indicators reflecting the coagulation function itself may have greater significance than the concentration of coagulation factors and related indexes. Indeed, activated partial thrombin time (APTT) is one of the independent risk factors for T2DM (14). Additionally, abbreviated APTT and elevated superoxide dismutase levels are linked in patients with T2DM (15). Interestingly, in our previous research, we found a correlation between oxidative stress-related uric acid levels and DPN in patients with T2DM (16).

In general, the relationship between blood glucose control, coagulation function, and DPN among patients with diabetes is not yet fully understood. Considering the heterogeneity of diabetic patients and the complexity of coagulation function (including concentrations of fibrinogen and D-dimer, along with their respective indexes, as well as parameters directly reflecting coagulation function such as APTT, prothrombin time, thrombin time, etc.), as well as the relationship between plasma glucose control and coagulation function and DPN, this study aims to investigate whether the level of HbA1c, reflecting plasma glucose control in patients with T2DM, may be involved in DPN through coagulation function, especially APTT, which directly reflects coagulation function.

## 2 Methods

### 2.1 Experiment design and ethics

This cross-sectional study was conducted at the Department of Endocrinology, The First Affiliated Hospital of USTC. A total of 183 patients participated, all of whom met the standard criteria for T2DM. Prior to the start of the experiment, participants were fully informed about the clinical research procedures. Each volunteer provided informed consent, confirmed by their signature. Additionally, the study received approval from our institution’s Medical Research Ethics Committee (Approval No.: 2023-RE-013). This cross-sectional study adhered to the principles of the Declaration of Helsinki.

### 2.2 Inclusion and exclusion criteria

All participants in this study were diagnosed with diabetes and met the standards set by the World Health Organization (17). Specifically, we included patients diagnosed with T2DM. Specifically, the study included patients diagnosed with T2DM. The exclusion criteria were consistent with those from our previous study (11) and were as follows: a) other types of diabetes (such as type 1 diabetes, gestational diabetes, and specific subtypes); b) diabetes with acute complications; c) neuropathy caused by other diseases or medications; d) severe vascular conditions (such as venous embolism, lymphangitis) and disease with hematological disorders; e) drug-induced neurotoxicity; f) other unspecified conditions or medications that might influence neuropathy; g) a history of amputation; h) diagnosed thyroid disorders (excluding thyroid nodules); i) tobacco or alcohol abuse; j) any other unspecified conditions that could affect the results of neurophysiological assessments. The diagnosis of DPN was based on the Toronto Consensus criteria (7). Patients with T2DM who were diagnosed with DPN were assigned to the DPN group, while those who did not meet the criteria for DPN were assigned to the control group.

### 2.3 Clinical data

The study collected data on participants’ age, gender, height, weight, and duration of diabetes mellitus (DM). Information on medication use, particularly anticoagulants, was also recorded. Body mass index (BMI) was calculated using the formula weight (kg)/height (m)². Fasting plasma glucose (FPG) levels were measured using the dry chemical method, and HbA1c levels were determined by microcolumn ion-exchange chromatography. Blood samples were used to measure lipid profiles, including high-density lipoprotein cholesterol (HDL-C), low-density lipoprotein cholesterol (LDL-C), total cholesterol (TC), triglycerides (TG), and very low-density lipoprotein cholesterol (VLDL-C), as well as fasting C-peptide (FCP). The Homeostatic Model Assessment of Insulin Resistance (HOMA-IR) was calculated with the formula 1.5 + (fasting blood glucose [FBG] in mmol/L) × (fasting C-peptide [FCP] in pmol/L)/2800. These measurements were performed at The First Affiliated Hospital of USTC, Center Laboratory, for medical purposes, and the data will be subjected to further analysis.

### 2.4 Neurophysiological tests

Neurophysiological examinations were performed on patients with T2DM in our hospital by trained staff in the Electrophysiology room of our hospital, using an electromyographic evoked potential meter according to the manufacturer’s instructions (Natus Neurology, USA). The examination room should be maintained at a temperature between 20-25°C. The room should provide comfortable lighting and remain quiet throughout the examination to ensure there are no disturbing noises. Nerve conduction velocity data were retrieved from the medical records of the T2DM patients involved in the study. The mean values of nerve conduction velocities from both sides were calculated for further analysis (18, 19).

### 2.5 Statistical methods

In this study, SPSS 26.0 (IBM Corp, NY, US) with PROCESS macro 4.1 was used to analyze the data. Variables with a normal distribution, such as LDL-C, median nerve motor conduction velocity, and the sensory conduction velocities of the ulnar and sural nerves, were reported as mean ± standard deviation. Differences in these variables between diabetic patients with and without DPN were compared using Student’s t-tests. Age, BMI, duration of diabetes, HbA1c, TG, TC, HDL-C, VLDL-C, fibrinogen, D-dimer, prothrombin time, thrombin time, APTT, and INR, as well as motor conduction velocities of the ulnar, radial, tibial, and common peroneal nerves, were described using the median and interquartile range. Differences in these variables between diabetic patients with and without DPN were assessed using the nonparametric Mann-Whitney U test due to their symmetric distribution. Gender and information regarding the use of anticoagulant drugs were expressed as percentages, and differences between T2DM patients with and without DPN were analyzed using the Chi-squared test since they were binary variables. To explore the relationships between DPN and HbA1c (or APTT), Pearson correlation or Partial correlation (adjusting for age, gender, duration of DM, and BMI) and binary logistic regression analyses were performed. Mediation analysis was conducted to assess the total, direct, and indirect effects of HbA1c on specific nerve conduction velocity, with APTT as a mediator. This involved breaking down the “total effect” into a “direct effect” (not mediated by APTT) and an “indirect effect” (mediated by APTT) and calculating the mediation effect as the indirect effect divided by the total effect, multiplied by 100%. The significance of the mediation effect was evaluated using bootstrap testing. An analysis was also conducted to determine whether APTT mediated the effect of HbA1c on DPN. Statistical significance was defined as P < 0.05 (20).

## 3 Results

### 3.1 Clinical data of T2DM patients

The clinical data of participants enrolled in this present study were summarized in **Table 1**. As showed, among these, 82 were identified with DPN, while 101 were determined to be free of DPN. As a cross-sectional study, although our patients were not strictly matched prior to inclusion, there were no statistically significant differences in age and gender between the two groups of patients (all *P >*0.05). Similarly, there were no differences in BMI and diabetes duration between the two groups (all *P >*0.05). Subsequent analysis of other clinical parameters revealed that, although the HbA1c levels were significantly higher in the DPN group compared to the control group (*P <*0.001), there were no differences between the two groups in HOMA-IR and blood lipid profiles (including TG, TC, HDL-C, LDL-C, and VLDL-C) (all *P >*0.05). Since our study involved coagulation function, we specifically compared PT, INR, APTT, Fibrinogen, TT, and D-Dimer between the two groups. The results showed that, except for APTT, which differed between the two groups (*P* =0.031), there were no statistically significant differences in PT, INR, Fibrinogen, TT, and D-Dimer (all *P >*0.05). Additionally, to further minimize study bias, we analyzed the use of anticoagulants in both groups. The results showed that only six patients in each group were using anticoagulants, and the proportion of anticoagulant use did not differ between the two groups (*P >*0.05).

**Table 1:** Comparation of clinical parameters and neurophysiological test results between Control and DPN group.

	Control group (n=101)	DPN group (n=82)	p
Age (years)	58 (49.5-68)	58.5 (52.75-68.25)	0.959^b^
Female (n, %)	42, 41.58	37, 45.12	0.858^c^
BMI (m^2^/kg)	24.03 (22.58-25.74)	23.57 (21.45-25.92)	0.330^b^
Duration of DM (years)	10 (4-15)	10 (1-15)	0.990^b^
HbA1c (%)	8.0 (7.0-9.35)	9.6 (7.98-11.8)	0.000^b*^
HOMA-IR	2.69 (2.10-3.46)	2.83 (2.20-3.63)	0.418^b^
TG (mmol/l)	1.50 (1.12-2.32)	1.46 (1.13-2.29)	0.935^b^
TC (mmol/l)	4.46 (3.70-4.94)	4.56 (3.82-5.17)	0.608^b^
HDL-C (mmol/l)	1.04 (0.91-1.24)	1.08 (0.91-1.21)	0.282^b^
LDL-C (mmol/l)	2.65 ± 0.80	2.66 ± 0.77	0.890^a^
VLDL-C (mmol/l)	0.61 (0.43-0.79)	0.70 (0.47-0.95)	0.095^b^
Fibrinogen	3.22 (2.68-3.70)	3.13 (2.65-3.74)	0.587^b^
D-Dimer	0.22 (0.17-0.33)	0.25 (0.19-0.43)	0.518^b^
prothrombin time	12.40 (11.85-12.90)	12.25 (10.90-12.9)	0.682^b^
thrombin time	17.80 (17.00-18.35)	17.85 (17.40-18.50)	0.472^b^
APTT	34.40 (32.40-36.60)	33.05 (28.13-35.04)	0.031^b*^
INR	0.94 (0.91-0.98)	0.93 (0.90-0.98)	0.806^b^
Anticoagulant drugs (n, %)	6, 5.94	6, 7.31	0.708^c^
Motor conduction			
Ulnar nerve (m/s)	55.15 (52.65-58.55)	53.30 (50.39-54.96)	0.003^b*^
Radial nerve (m/s)	63.50 (62.25-65.10)	63.00 (61.10-65.40)	0.330^b^
Median nerve (m/s)	56.23 ± 3.62	51.55 ± 4.24	<0.001^a*^
Tibial nerve (m/s)	45.95 (43.65-48.03)	41.32 (39.68-44.01)	<0.001^b*^
Common peroneal nerve (m/s)	44.40 (42.58-46.53)	41.18 (37.85-44.69)	<0.001^b*^
Sensory conduction			
Ulnar nerve (m/s)	55.69 ± 4.99	50.53 ± 6.00	<0.001^a*^
Radial nerve (m/s)	59.05 (54.92-63.83)	52.55 (48.68-57.40)	<0.001^b^
Median nerve (m/s)	56.00 (52.10-59.63)	45.38 (38.64-52.84)	<0.001^b*^
Sural nerve (m/s)	51.56 ± 5.42	46.46 ± 6.27	<0.001^a*^

a Student’s t test was employed for normally distributed variables.

b The Mann-Whitney U test was employed for asymmetrically distributed variables.

c The Chi-square test was employed for categorical variables.

*P<0.05.

DPN, diabetic peripheral neuropathy; BMI, body mass index; DM, diabetes mellitus; HBP, high bold pressure, HbA1c, Glycosylated hemoglobin; TG, triglycerides; TC, Total cholesterol; LDL-C, low-density lipoprotein cholesterol; HDL-C, high-density lipoprotein cholesterol; VLDL-C, very low-density lipoprotein cholesterol; PT, prothrombin time; INR, international rate; APTT, activated partial thrombin time; TT, thrombin time.

### 3.2 Difference of neurophysiological examination results in two groups

To objectively describe the extent of neuropathy, a neurophysiological assessment was conducted to measure the patients’ nerve conduction velocities. Intriguingly, except for the radial nerve (*P* = 0.330), all motor fibers, including the ulnar, median, and tibial nerves, exhibited reduced conduction velocities in T2DM patients suffering from DPN compared to those without DPN (as presented in the lower part of **Table 1**) (all *P* < 0.001). Additionally, all sensory fibers, including the ulnar, radial, median, and sural nerves, showed decreased conduction velocities in T2DM patients with DPN (all *P* < 0.001).

### 3.3 Correlation between HbA1c and nerve conduction velocity

To explore the link between HbA1c and DPN, a Pearson correlation analysis was conducted. Remarkably, the results revealed that HbA1c levels are not only negatively correlated with the motor fiber conduction velocities of the Ulnar, Tibial, Median, Tibial, and Common peroneal nerves, but also negatively associated with the sensory fiber conduction velocities of the Ulnar, Radial, Median, and Sural nerves (all P < 0.05). Additionally, these associations persisted even when analyzed by partial correlation, adjusting for age, gender, duration of DM, and BMI (all P < 0.05) (as demonstrated in **Table 2**).

**Table 2:** Pearson correlation between HbA1c and nerve conduction velocity.

	Model 1		Model 2	
	R	P	R	P
Motor conduction				
Ulnar nerve	-0.241	0.001^*^	-0.285	<0.001^*^
Radial nerve	-0.168	0.023^*^	-0.153	0.040^*^
Median nerve	-0.320	<0.001^*^	-0.391	<0.001^*^
Tibial nerve	-0.362	<0.001^*^	-0.439	<0.001^*^
Common peroneal nerve	-0.328	<0.001^*^	-0.388	<0.001^*^
Sensory conduction				
Ulnar nerve	-0.113	0.129	-0.160	0.032^*^
Radial nerve	-0.104	0.162	-0.085	0.260
Median nerve	-0.153	0.038^*^	-0.260	<0.001^*^
Sural nerve	-0.272	<0.001^*^	-0.297	<0.001^*^

*P<0.05. Model 1 showed person association between HbA1c and verve conduction velocity; Model 2 showed partial association between HbA1c and verve conduction velocity adjusting for age, gender, duration of DM, and BMI.

HbA1c, Glycosylated hemoglobin; DM, diabetes mellitus; BMI, body mass index.

### 3.4 Correlation between APTT and nerve conduction velocity

In order to clarify the relationship between APTT and DPN, a Pearson correlation analysis was conducted. Interestingly, the results showed that APTT levels are not only negatively correlated with the motor fiber conduction velocities of the tibial nerve, but also negatively associated with the sensory fiber conduction velocities of the median and sural nerves (all P < 0.05). Additionally, these associations persisted even when analyzed by partial correlation, adjusting for age, gender, duration of DM, and BMI (all P < 0.05). Although there is no statistically significant correlation between the sensory fiber conduction velocity of the ulnar nerve and APTT when analyzed by Pearson correlation, an association is observed after adjusting for age, gender, duration of DM, and BMI (analyzed by partial correlation) (as depicted in **Table 3**).

**Table 3:** Pearson correlation between APTT and nerve conduction velocity.

	Model 1		Model 2	
	R	P	R	P
Motor conduction				
Ulnar nerve	-0.112	0.132	-0.067	0.371
Radial nerve	0.057	0.444	0.051	0.502
Median nerve	0.009	0.900	0.052	0.487
Tibial nerve	0.190	0.010^*^	0.250	0.001^*^
Common peroneal nerve	-0.016	0.832	0.039	0.606
Sensory conduction				
Ulnar nerve	0.142	0.056	0.196	0.008^*^
Radial nerve	0.135	0.069	0.133	0.077
Median nerve	0.149	0.044^*^	0.171	0.022^*^
Sural nerve	0.213	0.004^*^	0.269	<0.001^*^

*P<0.05. Model 1 showed person association between APTT and verve conduction velocity; Model 2 showed partial association between APTT and verve conduction velocity adjusting for age, gender, duration of DM, and BMI.

APTT, activated partial thrombin time; DM, diabetes mellitus; BMI, body mass index.

### 3.5 Binary logistic regression for risk factors of DPN

**Figure 1:**
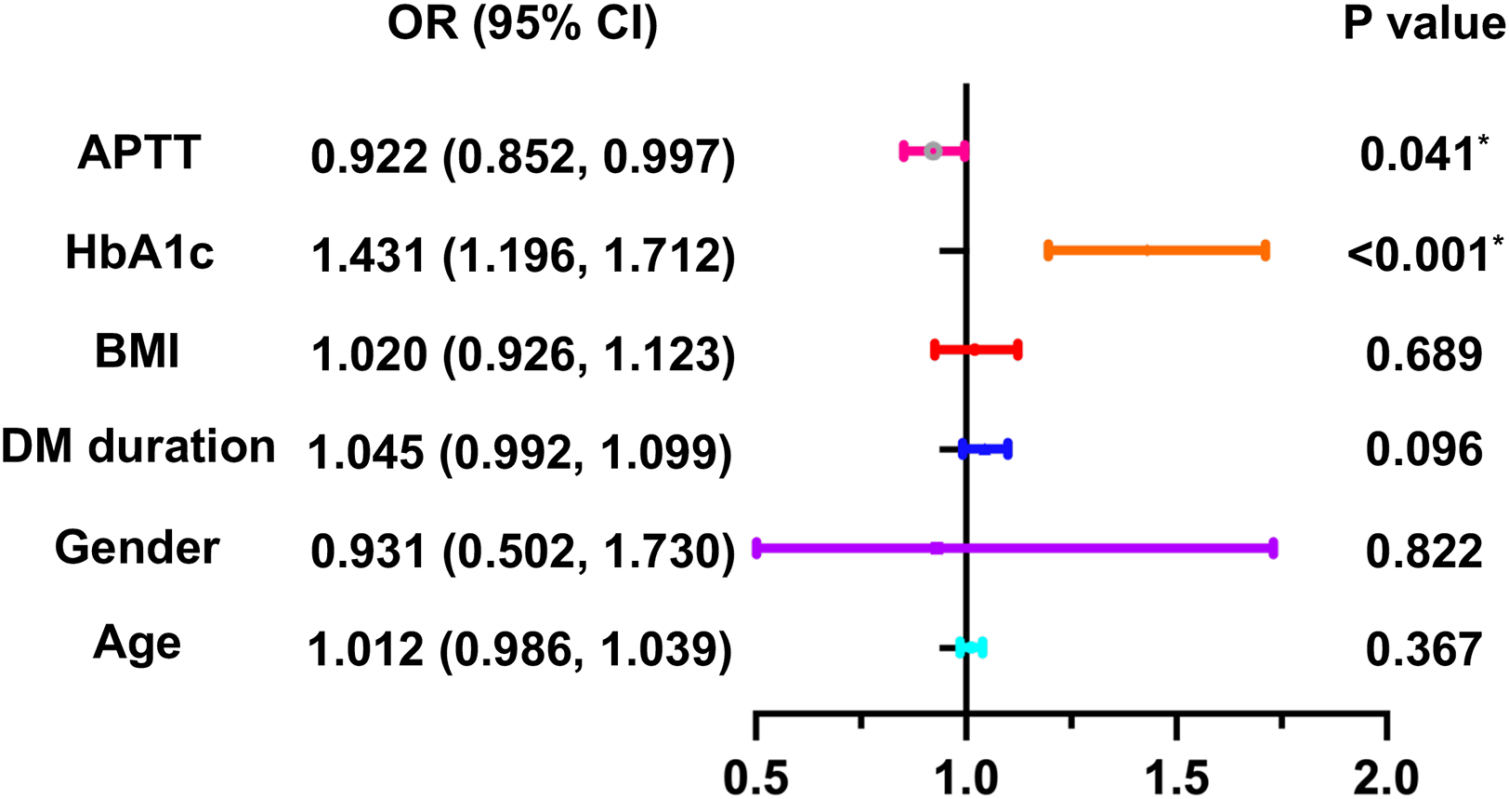
Binary logistic regression analysis for risk factors of DPN in T2DM patients. *P<0.05. DPN, diabetic peripheral neuropathy; T2DM, Type 2 diabetes mellitus; HbA1c, Glycosylated hemoglobin; APTT, activated partial thrombin time; BMI, body mass index; DM, diabetes mellitus.

In order to explore the risk factors of DPN in individuals diagnosed as T2DM, a binary logistic regression analysis was conducted. The results indicated that not only HbA1c, but also APTT were risk factors for DPN in volunteers diagnosed as T2DM (OR= 0.923 and 1.320, *P* = 0.001 and 0.041, respectively), as shown in **Table 4**. Additionally, HbA1c and APTT are still risk factors for DPN in patients with T2DM. after adjusting for age, gender, duration of DM, and BMI (OR= 0.922 and 1.431, *P* < 0.001 and 0.041, respectively) (**Figure 1**).

**Table 4:** Binary logistic regression analysis for risk factors of DPN in T2DM patients.

	p	OR	95% CI of OR	
HbA1c	0.001^*^	1.320	1.129	1.544
APTT	0.041^*^	0.923	0.855	0.997

*P<0.05.

DPN, diabetic peripheral neuropathy; T2DM, Type 2 diabetes mellitus; HbA1c, Glycosylated hemoglobin; APTT, activated partial thrombin time.

### 3.6 The medicating role of APTT in the association between HbA1c and DPN

**Figure 2:**
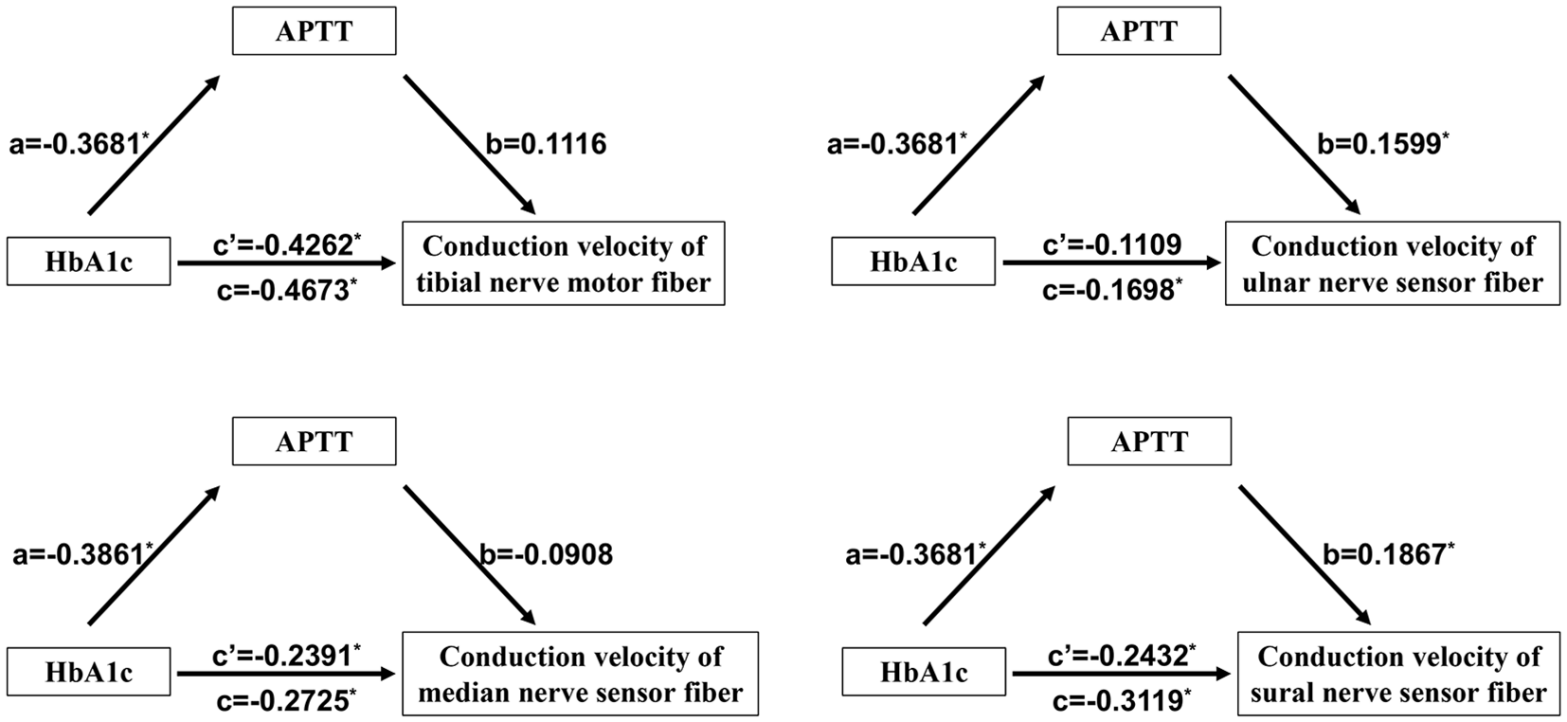
Mediating role of APTT in the relationship between HbA1c and DPN in T2DM patients. *P<0.05. APTT, activated partial thrombin time; HbA1c, Glycosylated hemoglobin; DPN, diabetic peripheral neuropathy; T2DM, Type 2 diabetes mellitus.

As previously mentioned, there is an association between the conduction velocities of motor fibers in the tibial nerve and sensory fibers in the tibial, median, and sural nerves, and APTT, as well as a correlation between these conduction velocities and HbA1c. Furthermore, both HbA1c and APTT are risk factors for DPN in patients with T2DM. We hypothesize that APTT may mediate the effect of uncontrolled plasma glucose on DPN. To investigate the mediating role of HbA1c in the effect of LDL-C on DPN, we conducted a mediation analysis using IBM SPSS Statistics version 26.0 with Process 4.1 for Mac (as described in the methods section). The results are presented in **Table 5** and **Figure 2**. APTT mediated the indirect effect of HbA1c on the conduction velocities of sensory fibers in both the ulnar nerve and sural nerve (95% CI: -0.3448, -0.0135; -0.3523, -0.0180) (**Table 5**). There is a mediating role of APTT in the association between HbA1c and the conduction velocities of sensory fibers in the ulnar nerve. Additionally, APTT also mediated the effect of HbA1c on the conduction velocities of sensory fibers in the sural nerve. APTT mediated the relationship between HbA1c and the conduction velocities of sensory fibers in the ulnar nerve or sural nerve by 34.66% [(-0.3681 × 0.1599)/-0.1698] or 22.03% [(-0.3681 × 0.1867)/-0.3119], respectively (**Figure 2**).

**Table 5:** Analysis the effect of HbA1c on nerve conduction velocity medicated by APTT.

Path route	Effect	Effect value	SE	95% CI	
				lower	upper
HbA1c → APTT → Tibial nerve motor fibers	Direct	-0.8074	0.1445	-1.0926	-1.5221
	Indirect	-0.0778	0.0591	-0.2206	0.0142
HbA1c → APTT →Ulnar nerve sensory fibers	Direct	-0.2902	0.2176	-0.7196	0.1392
	Indirect	-0.1540	0.0835	-0.3448	-0.0135
HbA1c → APTT →Median nerve sensory fibers	Direct	-0.8725	0.2959	-0.2886	-0.2391
	Indirect	-0.1219	0.1166	-0.3403	0.1309
HbA1c → APTT →Sural nerve sensory fibers	Direct	-0.6681	0.2174	-1.0970	-0.2432
	Indirect	-0.1888	0.0855	-0.3523	-0.0180

HbA1c, Glycosylated hemoglobin; APTT, activated partial thrombin time. → means the effect of HbA1c on Nerve via APTT.

## 4 Discussion

According to estimates, the prevalence of diabetes is increasing year by year both globally (21) and in mainland China (22). In addition to hyperglycemia itself and the acute complications it causes, the chronic complications of diabetes not only severely impact patients’ quality of life and lifespan but also impose a significant financial burden on both society and individuals due to the enormous medical expenses. The pathogenesis of complications, including diabetic peripheral neuropathy, involves chronic hyperglycemia (1, 2), insulin resistance (23, 24), chronic metabolic inflammation (25, 26), oxidative stress (27, 28), demyelination (29), and coagulation dysfunction (3–5) etc. Among these, chronic hyperglycemia is the most extensively studied in the context of diabetic peripheral neuropathy, while coagulation dysfunction has also been investigated to some extent, though research in this area is relatively limited.

For chronic hyperglycemia, the persistent presence of high plasma glucose is closely related to diabetes and DPN. Indeed, the duration of diabetes is known to be a risk factor for DPN in individuals with T2DM (30). Regarding the degree of hyperglycemia, as mentioned earlier, many studies have shown that elevated plasma glucose levels are associated with DPN. Actually, hyperglycemia is one of the most important factors related to diabetic complications (31, 32). In this study, we observed increased levels of HbA1c in T2DM participants with peripheral neuropathy. Additionally, we demonstrated that elevated levels of HbA1c are correlated with decreased conduction velocity in the motor fibers of Ulnar, Radial, Median, Tibial, and Common peroneal nerves, as well as sensory fibers of Ulnar, Median, and Sural nerves. These findings are consistent with a recent study, which indicated that elevated HbA1c levels are linked to DPN in patients with diabetes (33). In this present study, we not only identified a potential correlation between HbA1c and DPN but also quantitatively described the nerve conduction velocity in diabetic patients. This quantitative data was subsequently used for correlation analysis, regression analysis, and mediation analysis.

For coagulation function, we first compared fibrinogen, which is our primary concern. We found that there was no difference in fibrinogen levels between type 2 diabetes patients with and without peripheral neuropathy. The same was true for D-dimer levels. This finding is not consistent with previous studies, which have suggested that fibrinogen and D-dimer levels may be associated with DPN. Based on our review of the literature, we believe this discrepancy may be due to several factors as following. Although there is a prevalence of abnormal coagulation function among diabetic patients, their heterogeneity is substantial. In patients type 1 and type 2 diabetes mellitus, changes in coagulation function exhibit both similarities and distinct characteristics unique to each (34). Additionally, Fibrinogen levels in females with T2DM surpass those in males, and even after accounting for confounding variables, females still exhibit denser clots more resilient to fibrinolysis (35). Nonetheless, a separate study with a smaller sample size reported no alterations in fibrinogen levels among females with T2DM (36). When it comes to a younger group diagnosed with type 1 diabetes mellitus (under 30 years old), females exhibit an extended lysis time in comparison to males, whereas this distinction does not apply to an older demographic (37). When considering the influence of coagulation function on DPN, in addition to the concentrations of coagulation-related factors themselves, it was found that some calculated indexes (including fibrinogen/albumin, prealbumin to fibrinogen ratio, Fibrinogen function indexes) may hold promise as biomarkers for DPN (38–40). Except for concentrations of fibrinogen and its associated indexes, there is an association between fibrinogen gene polymorphisms and DPN in young patients with diabetes. Indeed, A allele and AA genotype of rs1800790 seem to be associated with DPN in young patients with diabetes (41). Indeed, other study had shown that genetic polymorphisms may play a role in neuropathy (42). In the correlation analysis, our study found differences in the relationship between our research variables and the conduction velocities of different nerve fibers. We believe this may be due to two potential factors. On the one hand, structural differences may exist between different types of nerve fibers (including variations in location or type). On the other hand, since our study is based on a small sample size, such differences may be subject to bias. This is one of the limitations of our study, and we must acknowledge it.

Since our research did not find any significant differences in fibrinogen and D-dimer levels between T2DM patients with and without DPN, we further compared other coagulation-related indicators. These indicators included APTT, prothrombin time, thrombin time, and international normalized ratio (INR), among others. Our research has revealed that patients with T2DM accompanied by peripheral neuropathy exhibit significantly shortened APTT. Shortened APTT is not only an independent risk factor for peripheral neuropathy in T2DM patients, but it is also negatively correlated with the nerve conduction velocity of several nerve fibers.

Given the similarities between HbA1c levels, which represent glycemic control, and APTT in relation to DPN, we hypothesize that APTT may mediate the effect of glycemic control on DPN. Indeed, studies suggest that diabetes may potentially affect coagulation function. This could be related to the advanced glycation end products that are produced in a state of chronic hyperglycemia (12–14). Therefore, in our subsequent research, we analyzed the mediating role of APTT in the impact of poor glycemic control on nerve conduction velocities. Intriguingly, the impact of elevated HbA1c levels on the conduction velocity of sensory fibers in the ulnar or sural nerves was partly mediated by APTT in individuals with T2DM. From a statistical perspective, we found that the effect of HbA1c on DPN is indeed mediated by APTT, with the mediation effect considered to be partial. To the best of our knowledge, this is the first study to examine the mediating role of APTT in the impact of HbA1c on DPN in patients with type 2 diabetes mellitus. In this study, we found that the mediating effect identified in our research is a partial mediation. Therefore, in addition to APTT, there may be other variables mediating the impact of HbA1c on DPN. Simultaneously, there may also be factors independent of HbA1c that influence APTT. These factors could include medications, inflammatory markers, and others. Furthermore, as a clinical study, we can only hypothesize the possibility of such mediation effects, but the specific mechanisms remain to be elucidated through further basic research. The underlying mechanisms may involve hyperglycemia-induced microcirculatory disturbances, endothelial cell damage, and other related pathways.

In general, previous studies have primarily focused on identifying the risk factors for DPN in diabetic patients. This study goes further by not only examining those risk factors but also exploring the relationship between HbA1c levels and specific neuronal damage, with a specific focus on nerve conduction velocities, APTT levels, and HbA1c levels. While earlier research has discussed the link between APTT, HbA1c, and DPN, this study is the first to highlight the mediating role of HbA1c in the effect of elevated LDL-C levels on the sensory fiber conduction velocity of the ulnar (and sural) nerve in individuals with T2DM. However, several limitations need to be acknowledged. This is a small-scale, cross-sectional study, so only associations, rather than causal relationships, between HbA1c (or APTT) and nerve conduction velocities can be established. This study preliminarily clarified the relationship among HbA1c, APTT, and DPN, with a particular focus on the potential mediating effects. Therefore, we did not further explore the potential of APTT as a diagnostic marker for DPN, although we do not rule out this possibility. Additionally, we must acknowledge that APTT could potentially serve as a target for preventive interventions in DPN in the future. In our research, we concentrated on peripheral neuropathy, as its onset may occur as early as the prediabetic stage. Consequently, we did not strictly limit the duration of diabetes in our inclusion criteria. Similarly, at the study’s design stage, we only required patients to have T2DM, without setting age restrictions. Although almost all patients included in our study were over 50 years old, we must acknowledge this as one of the limitations of our research. For blood glucose control indicators, in addition to HbA1c, other metrics such as fasting glucose, postprandial glucose, and fructosamine are also commonly used, each with its unique characteristics. However, since the focus of our study is on HbA1c, we did not analyze other indicators. Furthermore, most of our patients underwent continuous glucose monitoring, which not only reflects blood glucose levels but also provides insights into glucose variability. Despite its advantages, we prioritized HbA1c in this study and therefore did not delve deeply into continuous glucose monitoring data. Additionally, it is worth noting that there is a certain degree of correlation among these blood glucose control indicators, and statistical collinearity between these metrics and HbA1c could be a concern. Of course, the exclusion of these other indicators remains a limitation of our study, which we have acknowledged in the discussion section. Smokers were excluded from our study design due to prior research indicating a significant impact of smoking on diabetic neuropathy (43). Likewise, patients with a history of alcohol abuse were also excluded, given the potential link between alcohol consumption and DPN (44, 45). Nevertheless, it would be valuable to specifically investigate the effects of smoking and alcohol consumption on DPN. However, accounting for these factors, including the duration and quantity of smoking and drinking, is complex and falls outside the primary scope of this study. Additionally, we did not include patients with indirect tobacco exposure, despite evidence suggesting that second-hand smoke might affect the nervous system (46). In this study, we focused on exploring the potential impact of blood glucose control on coagulation function and DPN. However, we did not delve further into the potential underlying mechanisms behind this clinical research. This highlights the distinction between clinical research and basic research. While our study is clinical in nature, the lack of investigation into potential mechanisms is considered one of its limitations. Additionally, the mediating effect identified in our research is a partial mediation. Therefore, beyond APTT, other variables may also mediate the influence of HbA1c on DPN. At the same time, there may be other factors independent of HbA1c that affect APTT, including medications, inflammatory factors, and others. In our study, in addition to focusing on the potential impact of blood glucose control on coagulation function, we should also consider other confounders that may affect coagulation. These factors include underlying hematological disorders and medications that influence coagulation. We excluded patients with potential hematological disorders based on our study’s exclusion criteria. Regarding medications affecting coagulation, we accounted for them during the initial phase of the study. Relevant data on medication usage were collected during the original data collection phase. Among the included patients, we did not find any significant difference in the usage rate of such medications between T2DM patients with and without peripheral neuropathy. To some extent, this mitigates the potential impact of coagulation-related medications on the study results. However, we must acknowledge the limitations of our research. Although we recorded whether patients used relevant medications, we did not account for the specific types of drugs, their dosages, or the cumulative duration of their use. This represents one of the limitations of our study.

## 5 Conclusion

In conclusion, elevated HbA1c levels have been shown to affect the sensory fibers of the ulnar and sural nerves in diabetic patients, and this effect is partially mediated by reduced APTT levels. Research indicates that the impact of high HbA1c on these nerves may depend on decreased APTT levels in individuals with T2DM. In clinical practice, focusing solely on controlling HbA1c is insufficient when addressing DPN. It’s crucial to also consider coagulation factors, particularly APTT, when evaluating the condition. Since our study did not further explore APTT as a biomarker—such as through ROC curve analysis, determination of cut-off points, and calculations of sensitivity and specificity—we cannot yet describe it as a definitive biomarker. Although our current findings do not establish APTT as a definitive biomarker, it remains a potential candidate. In fact, it may hold promise for future clinical practice, where stratified management of patients based on different APTT levels could be feasible.

## Data Availability

The raw data supporting the conclusions of this article will be made available by the authors, without undue reservation.
